# Long non-coding RNAs: novel regulators of cellular physiology and function

**DOI:** 10.1007/s00424-021-02641-z

**Published:** 2021-11-18

**Authors:** James A. Oo, Ralf P. Brandes, Matthias S. Leisegang

**Affiliations:** 1 Institute for Cardiovascular Physiology, Goethe University, Theodor-Stern-Kai 7, 60590 Frankfurt, Germany; 2 German Centre of Cardiovascular Research (DZHK), Partner Site RheinMain, Frankfurt, Germany

**Keywords:** Long non-coding RNA, Physiology, Pathophysiology, ncRNA

## Abstract

Long non-coding RNAs were once considered as “junk” RNA produced by aberrant DNA transcription. They are now understood to play central roles in diverse cellular processes from proliferation and migration to differentiation, senescence and DNA damage control. LncRNAs are classed as transcripts longer than 200 nucleotides that do not encode a peptide. They are relevant to many physiological and pathophysiological processes through their control of fundamental molecular functions. This review summarises the recent progress in lncRNA research and highlights the far-reaching physiological relevance of lncRNAs. The main areas of lncRNA research encompassing their characterisation, classification and mechanisms of action will be discussed. In particular, the regulation of gene expression and chromatin landscape through lncRNA control of proteins, DNA and other RNAs will be introduced. This will be exemplified with a selected number of lncRNAs that have been described in numerous physiological contexts and that should be largely representative of the tens-of-thousands of mammalian lncRNAs. To some extent, these lncRNAs have inspired the current thinking on the central dogmas of epigenetics, RNA and DNA mechanisms.

## LncRNA characteristics and classification

**Figure 1:**
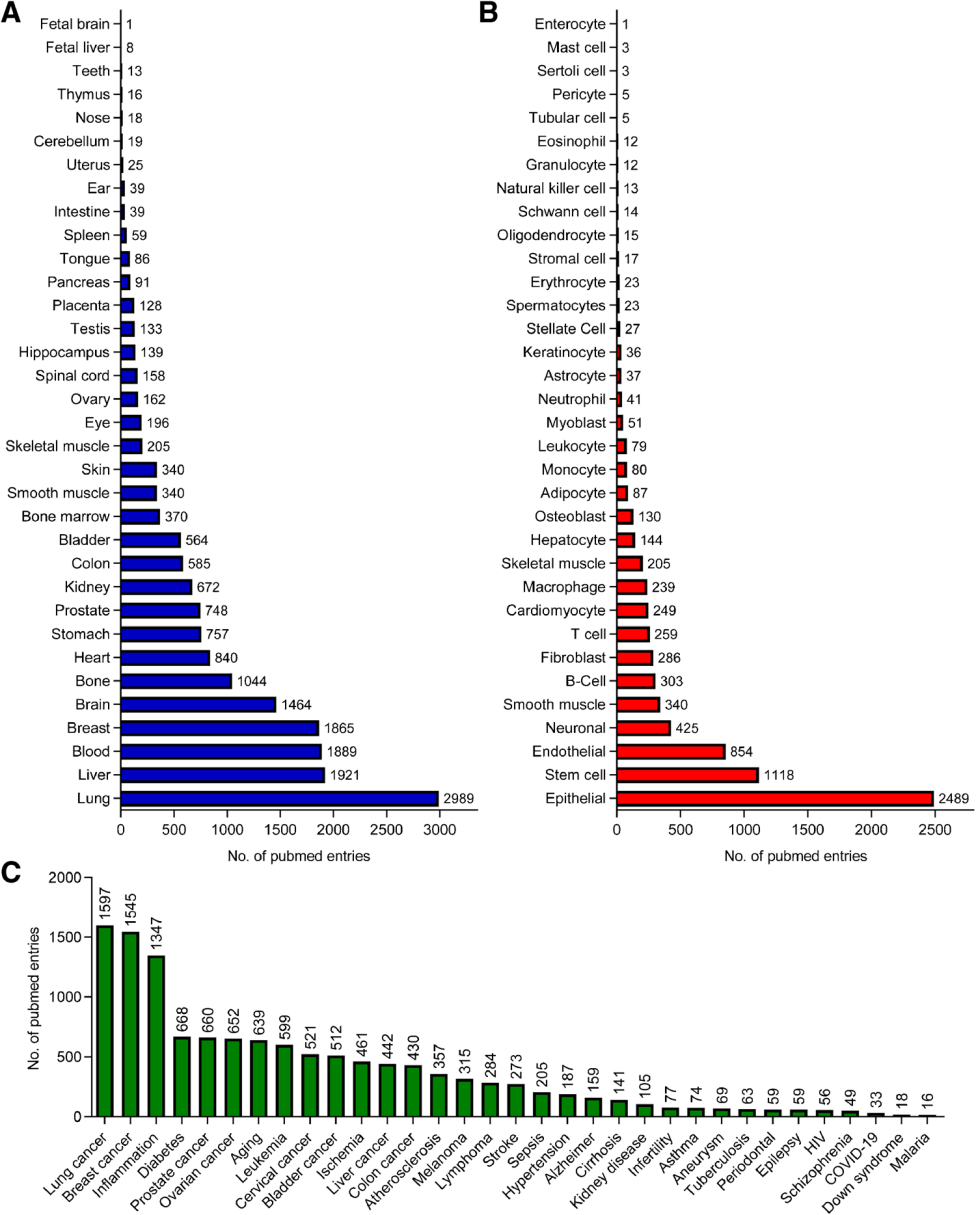
Publications about lncRNAs are existing in many different physiological areas. **A**–**C** Number of pubmed entries since 2008 for lncRNA and the individual tissue (**A**), cell type (**B**) or disease (**C**). PubMed searches were performed with the following terms: (“LncRNA” AND “search term”) OR (“Long non-coding RNA” AND “search term”) OR (“long non coding RNA” AND “search term”), as of date 5th October 2021.

The advancement of next generation sequencing and bioinformatic techniques in the last 20 years has led to the detection of genome-wide transcriptional events within non-coding regions and the subsequent discovery of thousands of long non-coding RNAs (lncRNAs). Countless studies have already revealed that lncRNAs are relevant for many physiological and pathophysiological processes (Fig. 1A–C) and that these can often be connected to the respective lncRNA molecular mechanisms of action.

**Figure 2:**
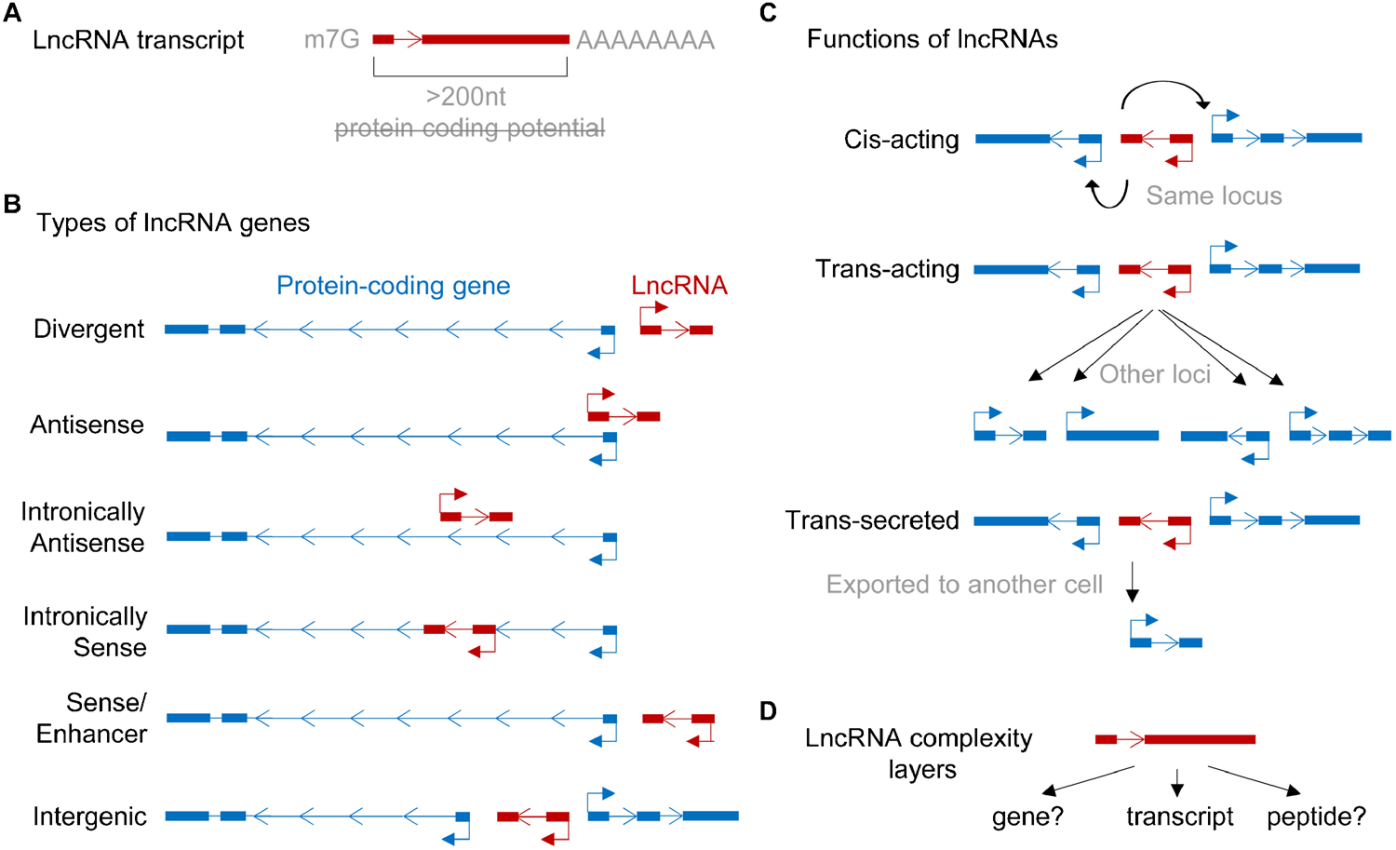
Research outlines and lncRNA characteristics. **A** LncRNA transcripts are defined as non-coding RNAs longer than 200nt apparently lacking protein coding potential. Typically, the majority of lncRNAs are mRNA-like RNAs harbouring a 5′Cap and a polyA tail. **B** Genomic location of lncRNA genes. **C** LncRNAs can act *in cis* to regulate the immediate locus from which the lncRNA was transcribed, *in trans* to function elsewhere in the cell or *trans*-secreted. **D** For some lncRNA genes, functions on their gene itself, their transcript or peptide are known increasing the layer of complexity for their mode of operations.

Only a small fraction of the human transcriptome is translated into proteins since the majority of RNA transcripts are non-coding. These non-coding RNAs can be further divided into small non-coding RNAs, such as miRNAs, tRNAs, snoRNAs and snRNAs, and long non-coding RNAs (lncRNAs) which are longer than 200 nucleotides (Fig. 2A) [61]. Current estimates place the number of human lncRNAs at around 100,000 according to the NONCODE (v6) database [78]. Despite their lack of coding potential, lncRNAs share multiple features with mRNAs including (1) RNA polymerase II–mediated transcription regulated by common epigenetic marks such as tri-methylation of lysine 4 of histone 3 (H3K4me3); (2) a 7-methyl guanosine (m7G) 5′-Cap and Poly-A tail; and (3) splicing of multi-exonic transcripts, albeit less efficiently for lncRNAs than for mRNAs [61]. lncRNAs can be found anywhere in the cell but with the majority being localised to the nucleus; potentially a consequence of inefficient splicing events [56]. In contrast to mRNAs, many lncRNAs are relatively lowly expressed, less well conserved evolutionarily and highly cell type– or tissue-specific [61]. This difference is supported by studies demonstrating that lncRNA promoters contain fewer transcription factor (TF) binding motifs and TF binding events. Additionally, it is believed that the lower abundance of lncRNA transcripts cannot be explained by RNA degradation alone [56]. LncRNAs also form secondary and tertiary structures and contain functional RNA elements and nuclear localisation sequences, which are assumed to be important primarily for gene regulation [56, 67]. These regulatory motifs and higher order structures enable an exceedingly diverse range of lncRNA functions.

An official and appropriate classification that encompasses all lncRNAs does not yet exist [46]. One proposed method of lncRNA classification is based on their genomic position relative to other genes, such as protein-coding genes (Fig. 2B): (1) Divergent and antisense lncRNAs either overlap or are in close proximity to a sense gene and are localised on the opposite DNA strand; (2) intronic lncRNAs are transcribed from the intron of a sense or antisense gene; and (3) intergenic lncRNA genes which do not overlap other genes [46]. Each of these genomic arrangements are able to produce molecular and physiologically relevant lncRNAs. Other means of classification can be based on lncRNA modes of action and regulation, which include but are not limited to target gene regulation (by *cis*- or *trans*-acting lncRNAs, Fig. 2C) [46], molecular role (e.g. competitive endogenous RNAs [63], enhancer RNAs [32], architectural RNAs [8]), their transcriptional regulation (e.g. stress-induced promoter-associated antisense lncRNAs [20], damage-induced lncRNAs [50]) or their physiological relevance (e.g. Angio-LncRs [74]). More recently, it has been suggested that the process of transcription itself could have an important function independent of the lncRNA transcript produced from that transcriptional activity. For example, the locus could be part of a 3D nuclear construct permissive to chromatin environment and gene regulation at the neighbouring locus [1]. Similarly, some lncRNAs may not be entirely non-coding and, despite their low coding potential, may give rise to small functional peptides (so-called micropeptides) while retaining an independent RNA function (Fig. 2D) [61]. This highlights that our understanding of the lncRNA landscape is subject to change and that an appropriate method of classification encompassing all lncRNAs remains a challenge.

## The diverse functions of lncRNAs

LncRNAs usually enact their functions by interacting with proteins, metabolites, DNA or even other RNAs. Additionally, important regulatory elements may be embedded within the gene body of lncRNA genes where transcriptional activity can influence genome structure, chromatin accessibility and neighbouring gene activity [1]. lncRNAs themselves are highly modifiable with many different “post-transcriptional” modifications reported so far, such as N6-methyladenosine (m6A), pseudouridine (Ψ), 5-methylcytosine (m5C) and N1-methyladenosine (m1A) [14, 15, 40, 49, 60, 77], where m6A for example can influence RNA structure to alter protein interactions [42] or lncRNA functions [43]. LncRNAs have been shown to be involved in many cellular processes, mainly in transcriptional regulation, post-transcriptional regulation (e.g. splicing), cell organellar and structural organisation and genome integrity [61]. The mechanisms of gene expression regulation by lncRNAs are particularly diverse since lncRNAs bind other molecules in abundance. This allows for multiple mechanisms whereby lncRNAs permit or inhibit the interactions between these molecules; for example the recruitment or decoying of chromatin remodelling complexes, proteins mediating histone modifications, transcription factor binding to gene regulatory regions or the interaction of RNA with DNA that leads to R-Loop or triplex formation [61]. This not only impacts on transcription but also on genome stability. LncRNAs frequently serve as scaffolds as in the case of several forms of nuclear condensates, which are membraneless RNA–protein compartments [61]. Post-transcriptional functions of lncRNAs include the interference of mRNA splicing, turnover, decay and translation. LncRNAs can also affect cellular function through interaction with other ncRNAs, such as miRNAs. This so-called competitive endogenous RNA function often leads to the protection of miRNA-targets [61]. Finally, the homeostasis of organelles such as exosomes and mitochondria has even been linked to lncRNAs [61]. Taken together, these diverse means of regulation and the mechanisms through which lncRNAs subsequently enact their functions allow for the fine-tuning and regulation of cellular processes that have consequences for many physiological processes.

## LncRNAs are physiologically relevant

Despite the fact that most lncRNAs are so far uncharacterised, there already exists an exhaustive list of lncRNAs that have been investigated across almost all physiological systems. Bioinformatic databases, as reviewed in [53], or RNA analysis tools, such as the RNA atlas [45] (R2: Genomics Analysis and Visualization Platform (http://r2.amc.nl)), provide an abundance of lncRNA data which can be exploited by researchers working in many different areas of physiology.

**Figure 3:**
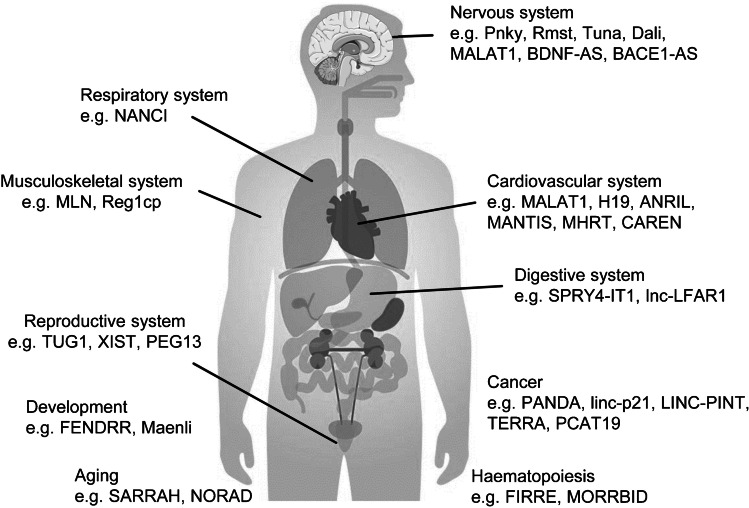
lncRNAs in various physiological systems and processes. LncRNAs have been shown to be fundamental in almost all physiological systems and processes. Example lncRNAs are provided for the major physiological systems and for a select number of general physiological processes to highlight the ubiquitous nature of lncRNAs.

Here, we will present examples of highly physiologically relevant lncRNAs and describe their mechanisms of action (Fig. 3). It should be stressed that we cannot cover all physiological processes and systems in one review and instead select a handful of lncRNAs that should be largely representative of the functions of this class of molecules. Additionally, many of these lncRNAs are involved in pathophysiological processes that have, for the most part, been historically characterised from a protein perspective. Finally, this review should also exemplify the strong influence that single lncRNAs can have on disease outcome: knowledge that can hopefully provide a platform for future basic and therapeutic RNA research.

### Loss of expression studies revealed that lncRNAs play critical roles in vivo

As early as 2013, a study with 18 lncRNA knockout mouse strains revealed that lncRNAs are important for viability and are involved in the development of lungs and the cerebral cortex. Peri- and postnatal lethal phenotypes were observed in lncRNA *Fendrr* (*FOXF1 Adjacent Non-Coding Developmental Regulatory RNA*), *Peril*, and *Mdgt* mutant strains and growth defects were reported for *linc–Brn1b* and *linc–Pint*. *Fendrr*^*−/−*^ neonates displayed defects in multiple organs. *Linc–Brn1b*^*−/−*^ mutants showed distinct abnormalities in the generation of upper layer II–IV neurons in the neocortex [58]. Lai et al. analysed 20 different lncRNA knockouts with a variety of phenotypes in mice, ranging from perinatal lethality to defects associated with premature aging and morphological and functional abnormalities in the lungs, skeleton and muscle [35]. An increased expression with age of the lncRNA *Lincpint* was observed in parallel with a reduction in body weight, probably due to reduced total body fat and lower femur bone mineral density, and the development of lordokyphosis. Knockout of *Fendrr* led to an abnormal lung morphology, *Hotair* knockout mice displayed a homeotic transformation in the 4th caudal vertebra and *Hottip* knockout showed hindlimb malformations [35]. In a large-scale cell culture analysis, as part of the FANTOM6 project, Ramilowski et al. knocked down 194 lncRNAs with at least two antisense oligonucleotides in human dermal fibroblasts and quantified cellular growth, morphological changes and transcriptomic responses with Capped Analysis of Gene Expression (CAGE) to measure the molecular phenotype. The authors observed that around 30% of the lncRNAs were associated with cell growth and morphological changes [54].

### X-chromosome inactivation, the first paradigm for a function of a lncRNA

*X-inactive specific transcript (XIST)* was one of the earliest described lncRNAs and, as such, studies on this lncRNA have provided an invaluable framework for research in the lncRNA field. *Xist* is responsible for X-chromosome inactivation; a process which achieves dosage compensation of the sex chromosomal genes between females and males. Both X chromosomes, the Xa (active) and Xi (inactive) chromosomes, contain the *XIST* gene, with the Xi gene initiating X-chromosomal inactivation during early development. *Xist* is transcribed and spreads in *cis* across the X-chromosome to coat Xi, but not Xa. The lncRNA triggers gene silencing by recruiting chromatin modifying factors including the polycomb-repressive complex 2 (PRC2), which results in a huge structural reorganization of the X-chromosome [17, 44].

lncRNA loci can be functionally highly complex as demonstrated in a study by Lewandowski et al. The authors identified that the widely expressed and highly conserved *Tug1* lncRNA is important for male fertility. *Tug1*-knockout mice were sterile with underlying defects in spermatogenesis as indicated by a low sperm count and abnormal sperm morphology. Molecular characterisation revealed that *Tug1*, however, functions beyond its role as a lncRNA: the locus acts as a *cis*-DNA repressor regulating neighbouring gene expression, whereas the lncRNA itself has a *trans*-regulatory function. Furthermore, the overexpression of an evolutionarily conserved open reading frame of *Tug1* encoded a protein identified to be important for mitochondrial membrane potential [39].

Mice lacking the 3′ half of paternally expressed gene, *Peg13*, which is part of a complex of imprinted genes on chromosome 15 in mice, showed distinct behavioural differences: they prefer to associate with their own sex after losing interest in the opposite sex. They also develop a higher level of anxiety, lowered activity and curiosity, and a deficiency in pup retrieval behaviour. The authors analysed whole-brain RNA of 16-week-old *Peg13*-deficient mice and revealed that expression of genes involved in the serotonergic system, formation of glutamatergic synapses, olfactory processing, and estrogen signalling and several others of the imprinted genes on chromosome 15 were changed. It was concluded that *Peg13* is part of a regulatory network that governs the female–male differentiation of the brain, as well as the neurobiology of social interactions [31].

### Developmental processes are dependent on lncRNAs

LncRNAs are also known to be central in developmental processes where their altered regulation often promotes disease. Grote et al. identified the lateral mesoderm-specific lncRNA *Fendrr* which regulates the development of the heart and body wall of developing mouse embryos [21]. In a similar manner as many other lncRNAs, *Fendrr* binds chromatin-associated protein complexes, specifically PRC2, and recruits it to genes where PRC2 deposits Histone3 Lysine27 trimethylation (H3K27me3) marks, leading to transcriptional repression. Concomitantly, *Fendrr* represses the Trithorax group/mixed lineage leukaemia complex (TrxG/MLL) at the same gene targets to prevent H3K4me3 deposition, normally associated with gene activation. This work highlights the cell-type specificity yet crucial roles of lncRNAs, where their perturbation can have drastic consequences on fundamental physiological processes such as organ development.

**Figure 4:**
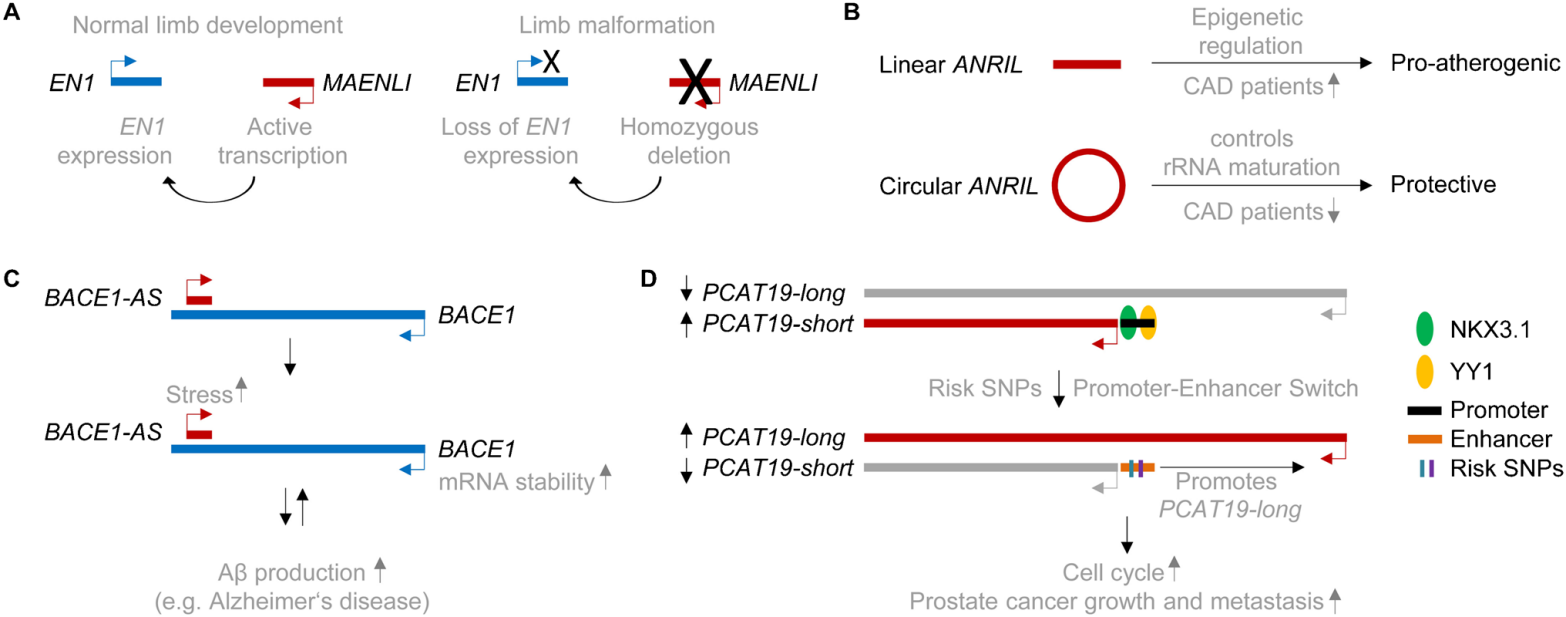
*MAENLI*, *ANRIL*, *BACE1-AS1* and *PCAT19* as examples of physiologically relevant lncRNA mechanisms. **A** For normal limb development, transcriptional activity of the *MAENLI* locus itself is required to activate *EN1* expression. Homozygous loss or deletion of the *MAENLI* locus abolish its transcriptional activity leading to the loss of *EN1* expression and limb malformation. **B** Linear *ANRIL* expression is increased in patients with coronary artery disease (CAD) patients and has pro-atherogenic functions through epigenetic rearrangements leading to altered expression of genes involved in atherosclerosis. Circular *ANRIL*, whose expression is decreased in CAD patients, is important for controlling rRNA maturation to protect from over-proliferation of vascular cells. **C** LncRNA *BACE1-AS* increases the mRNA stability of *BACE1*, resulting in an increased protein level of the β-secretase, which produces amyloid beta 1-42. This in turn activates *BACE1-AS* expression in a positive feedback loop. **D** *PCAT19* has two isoforms, a long and a short isoform. The short form is dominating and promoted by binding of the transcription factors NKX3.1 and YY1 to the short isoform promoter. Two risk SNPs for prostate cancer are located within the promoter of *PCAT19-short*, preventing NKX3.1 and YY1 from binding. The SNP-affected *PCAT19-short* promoter switches to an enhancer, promoting the expression of *PCAT19-long*. *PCAT19-long* activates transcription of genes involved in cell cycle and the growth and metastasis of prostate cancer cells.

*MAENLI* (*master activator of engrailed-1 in the limb*) is a lncRNA whose transcriptional activity is important for the deposition of active histone marks (Fig. 4A). Its deletion causes a severe human Mendelian disease [2]. Allou et al. identified homozygous 27–63 kb deletions located 300 kb upstream of the engrailed-1 gene (*EN1*) on human chromosome 2 in patients with a severe, recessively inherited congenital limb malformation featuring mesomelic shortening, syndactyly and ventral nails (dorsal dimelia). These deletions led to the loss of *En1* expression in the limbs of mice and a similar phenotypic outcome. Interestingly, they identified an unknown limb-expressed lncRNA within the deleted region, which they termed *Maenli* and which is part of an *En1* topologically associated domain, the central locus control element during embryonic limb development. The *Maenli* locus itself is essential to drive limb-specific *En1* activation in *cis* simply through its transcriptional activity. Mechanistically, *Maenli* transcription led to the deposition of H3K4me3 epigenetic marks on the *En1* and *Maenli* loci and the surrounding regulatory landscape. A similar effect on limb malformation was also seen after depletion of lncRNA *Hottip* in mice [35] and chicks [68], where Wang et al. showed that active chromatin of the 5′ *HOXA* cluster was controlled by *Hottip* RNA [68].

### Haematopoiesis and the immune system are controlled by lncRNAs

LncRNA *FIRRE* is a *trans*-acting lncRNA that regulates lymphopoiesis. Mice with *Firre* mutations have been shown to exhibit cell-specific lymphocyte phenotypes displayed by a reduction in the abundance of CD4 and CD8 T cells. Moreover, upon exposure to lipopolysaccharide, mice overexpressing *Firre* exhibited increased levels of the pro-inflammatory cytokines TNFα, IL12-p40, and MIP-2 and impaired survival [38].

*Morrbid* is a *cis*-acting lncRNA tightly controlling the lifespan of neutrophils, eosinophils and classical monocytes in response to pro-survival cytokines in mice. Mechanistically, the lncRNA promotes the enrichment of PRC2 and H3K27me3 at the promoter of its neighbouring gene, the pro-apoptotic gene *Bcl2l11* (*BIM*), which leads to repression of transcription. The lncRNA was found to be upregulated in individuals with hypereosinophilic syndrome, which is characterised by a persistently elevated eosinophil count [34].

### LncRNAs associated with aging

Aging is one of the main risk factors for numerous diseases. Trembinski et al. identified the conserved lncRNA *Sarrah* (*SCOT1-antisense RNA regulated during aging in the heart*), also known as OXCT1-AS1, as downregulated in aged mice and infarcted hearts [64]. Loss of the lncRNA impaired contractile force development in human engineered heart tissue. *Sarrah* was responsible for cardiomyocyte survival as its silencing led to apoptosis while its overexpression in mice improved their recovery from acute myocardial infarction. Mechanistically, the authors suggested that *SARRAH* forms RNA-DNA triplexes at gene promoters, which were downregulated after *SARRAH* silencing. An induction of NRF2 and the binding of CRIP2 and p300 facilitated transcriptional activation of *SARRAH* target genes.

Another lncRNA involved in aging is NORAD, which protects the genome by reducing the activity of PUMILIO proteins. NORAD depletion leads to overactivation of PUMILIO proteins with augmented repression of a program of target mRNAs that includes key regulators of mitosis, DNA repair, and DNA replication. Dysregulation of these genes could result in genomic instability in *Norad*-deficient cells reflected by faster aging in animals. Such a relationship is putatively seen since PUMILIO levels increase with age while those of NORAD decrease [48]. The investigation of the physiological function of lncRNA *Norad* by Kopp et al. revealed that Norad depletion led to a degenerative phenotype characterised by increased alopecia, gray fur, kyphosis and aging-associated pathologies within the central nervous system, which is the consequence of genomic instability and mitochondrial dysfunction, explained by PUMILIO2 overexpression [33].

## LncRNAs and their function in individual organ systems

### The cardiovascular system

Cardiovascular disease encompasses a diverse range of pathologies that make it the number one cause of death worldwide [70]. It is therefore unsurprising that many lncRNAs have been studied in the context of cardiovascular physiology and pathophysiology.

The lncRNA *MALAT1* is one of the few very highly expressed lncRNAs. Initially identified as a cancer biomarker [30], *MALAT1* has diverse roles in multiple different cancer types [62]. In the cardiovascular system, it was shown to be associated with atherosclerotic lesion formation in mice and with human atherosclerotic disease [10]. Reduced levels of *Malat1* had pro-atherosclerotic effects, which resulted from an increased accumulation of haematopoietic cells at the murine carotid artery vessel wall [10]. In failing hearts of mice, pigs and humans, expression of the lncRNA *H19* was reduced and an *H19* vector–based, cardiomyocyte-directed gene therapy was able to attenuate heart failure [66].

Multiple studies have been performed on the Chr9p21 locus, where numerous CAD risk SNPs accumulated within the gene encoding the lncRNA *ANRIL* (*antisense non-coding RNA in the INK4 locus*). *ANRIL* is an example of a lncRNA locus transcribing multiple isoforms, two of which are highly physiologically relevant: a linear lncRNA and a circular RNA with opposing functions (Fig. 4B). Linear *ANRIL* has pro-atherogenic effects involving mechanisms with Alu elements important for epigenetic gene regulation; however, the circular *ANRIL* isoform has putative protective functions which are mediated by inhibition of circular ANRIL’s interaction partner Pescadillo Ribosomal Biogenesis Factor 1 (PES1), a member of the PeBoW (Pes1, Bop1 and WDR12) complex. Inhibition of PeBoW leads to defects in rRNA maturation, increased nucleolar stress and activation of p53, which inhibits cell proliferation and increases apoptosis, whereas linear ANRIL confers overproliferation [26].

*MANTIS* is an example of a nuclear scaffolding lncRNA having a critical function in endothelial cells. Its depletion leads to a loss of the ability of endothelial cells to align in the direction of flow. Moreover, *MANTIS* is required to promote angiogenesis, limit inflammatory gene expression and maintain angiogenic capacity [36, 37]. Consequently, knockdown of *MANTIS* increased the adhesion of monocytes to endothelial cells in an ICAM-1-dependent manner. Statin therapy induced *MANTIS* in vitro and in patients with carotid artery disease. *MANTIS* also maintained the pleiotropic responses of endothelial cells to atorvastatin. Central to these effects is the interaction of *MANTIS* with the SWI/SNF chromatin remodelling complex member, BRG1. MANTIS guides BRG1 to its different effector genes, resulting in activation of pro-angiogenic gene expression and inhibition of inflammatory gene expression in endothelial cells [37].

LncRNA *Mhrt* is also connected to the chromatin remodeller BRG1. The lncRNA functions to limit cardiac hypertrophy by binding to and inhibiting the helicase domain of BRG1 to create a transcriptional feedback loop [22]. The inhibition of DNA-binding proteins is a common lncRNA mechanism of action that has profound effects on pathophysiological outcome.

The lncRNA *Caren* (*Cardiomyocyte-enriched transcript*) is a prime example of a cytoplasm-enriched lncRNA with a pivotal role in the development of a highly complex disease. It protects against the development of heart failure in pressure-overloaded hearts by inhibiting Hint1. Hint1 is a tumour suppressor that activates the ATM kinase and the DNA damage response (DDR), a process that is prominent in cardiomyocytes of patients with heart failure. *Caren’s* protective effects are related to the inhibition of Hint1-mediated mitochondrial dysfunction and DDR signalling through ATM. At least in mice, the overexpression of *Caren* alone is sufficient to prevent heart failure [57].

### The nervous system

lncRNA studies in the central nervous system have revealed a striking specificity of lncRNA expression in nervous tissue. Neurogenesis, the differentiation of neural stem cells (NSC) to neurons is a highly complex but well-orchestrated process that involves multiple physiological, molecular and genetic pathways. It is becoming evident that lncRNAs provide an additional layer of molecular control and that this is particularly true in neurogenesis and the complex differentiation of the brain.

*Pnky* (previously called *lnc-pou3f2*) is an evolutionarily conserved and neural-specific lncRNA involved in embryonic and postnatal neurogenesis. *Pnky* interacts with the RNA splicing regulator PTBP1 to mediate the expression and alternative splicing of transcripts important for limiting neural differentiation and neurogenesis [55]. PTBP1 has the ability to drive brain tumour growth and invasiveness [18], exemplifying the importance of PTBP1 inhibition by *Pnky*. Other prominent lncRNAs such as *RMST* (rhabdomyosarcoma 2–associated transcript) [52], *TUNA* (*Tcl1* Upstream Neuron-Associated lincRNA) [41] and *Dali* (DNMT1-Associated Long Intergenic) [6] also function in neurogenesis by physically interacting with proteins important for the regulation of neural gene expression. lncRNAs have also been shown to function in other central nervous processes such as synaptogenesis. Expression of the Brain-Derived Neurotrophic Factor (BDNF), which is essential for neuronal differentiation, maturation and growth, is inhibited by the BDNF antisense lncRNA, *BDNF-AS* [51]. *Malat1*, mentioned above for its function in tumours and the cardiovascular system, also controls the expression of genes involved in synaptogenesis and synapse function [4]. Many of the lncRNAs mentioned here are highly cell-type specific. On the other hand, *MALAT1* is ubiquitously expressed and has been shown to perform fundamental tasks in many cell types.

Given their roles in neuronal differentiation and development, it stands to reason that lncRNAs are involved in diseases of the nervous system, like neurodegeneration. For example, a central protein in the development of Alzheimer’s disease (AD), β-secretase (BACE1), produces the β amyloid plaques that are a hallmark of AD pathophysiology [11]. Interestingly, BACE1 expression is regulated to some degree by its antisense transcript, *BACE1-AS* (Fig. 4C). Disease-promoting factors such as amyloid-beta 1-42 upregulate *BACE1-AS* which promotes BACE1 expression and BACE1 mRNA stability to produce more amyloid beta 1-42 in a positive feedback loop [16]. This opens the possibility for RNA therapeutics targeting *BACE1-AS* and therefore BACE1 expression, which itself has already been the focus of intense AD therapeutic research [9].

### The respiratory system

As mentioned above already, lncRNAs are required for tissue and organ development, and this is also true for lung tissue. The lncRNA NANCI (Nkx2.1-associated non-coding intergenic RNA) associates with and promotes the expression of the Nkx2.1 transcription factor, which is crucial for pulmonary development and homeostasis. Loss of NANCI itself is not sufficient to limit development, since it is normally inhibited by Nkx2.1 in a negative feedback loop; however, mutations which impact both NANCI and Nkx2.1 lead to severe lung degeneration [25].

The lncRNA TYKRIL (tyrosine kinase receptor-inducing lncRNA) was found to be strongly upregulated in the pulmonary arterial pericytes and smooth muscle cells (SMC) of patients with idiopathic pulmonary arterial hypertension (IPAH) [75]. TYKRIL was shown to promote proliferation under the hypoxic conditions that are characteristic of IPAH. Interestingly, knockdown of TYKRIL was found to increase p53 levels which consequently repressed platelet-derived growth factor receptor β (PDGFRβ), a known driver of SMC proliferation. As such, TYKRIL has a profound influence on the development of PAH. Many lncRNAs have been shown to interact with and regulate p53 [7]; however, TYKRIL is the first lncRNA demonstrated to mediate the p53/PDGFRβ axis specifically. This exemplifies the fact that individual lncRNAs are capable of mediating common RNA-binding proteins in a precise and distinct manner.

One study demonstrated that the lung tissue from smokers with chronic obstructive pulmonary disorder (COPD) expressed 120 upregulated and 43 downregulated lncRNAs compared to smokers without COPD [12]. Similarly, smokers without COPD expressed 87 upregulated and 244 downregulated lncRNAs compared to non-smokers. This study nicely illustrates that whole networks of lncRNAs can be differentially expressed in a cell-type- and condition-specific manner.

### The digestive system

The intestinal barrier includes the chemical mucosal and physical epithelial layers that allow for selective absorption of nutrients, ions and water into the bloodstream. Barrier integrity is upheld by the tight junction proteins which are tightly regulated through multiple signalling pathways; dysregulation of which predisposes to inflammatory bowel diseases and metabolic disorders. lncRNAs also play a role in the context of intestinal barrier integrity. For example, depletion of the lncRNA *SPRY4-IT1* disrupted barrier integrity by reducing the stability of mRNAs encoding tight junction proteins claudin-1, claudin-3, occludin and JAM-1 [71]. Similarly, the lncRNA *H19*, heavily studied in the cardiovascular and cancer fields, is also involved in barrier integrity. *H19* acts as a precursor for microRNA 675 (miR-675) which represses the expression and reduces the stability of tight junction proteins ZO-1 and E-cadherin through its interaction with the RNA-binding protein HuR [79].

Zhang et al. identified the multi-functional lnc-LFAR1 (liver fibrosis-associated lncRNA1) that promotes liver fibrosis, a disease characterised by extracellular matrix component accumulation in the liver leading to hepatic dysfunction. Silencing of lnc-LFAR1 impaired hepatic stellate cells activation, reduced TGFβ-induced hepatocytes apoptosis and attenuated the CCl_4_- and bile duct ligation-induced liver fibrosis in mice. The authors revealed that Lnc-LFAR1 has multiple functions organising Smad2/3 binding, phosphorylation and induction to promote liver fibrosis leading to the activation of the TGFβ and Notch pathways [76].

### Musculoskeletal system

By definition, lncRNAs should lack protein-coding potential. However, as in the case for the lncRNA *Tug1* mentioned above, a peptide coded by the Myoregulin (MLN) lncRNA was shown to be important for skeletal muscle performance. MLN KO mice had improved skeletal muscle exercise performance and muscle Ca^2+^ handling. The conserved micropeptide of the lncRNA, which encodes a transmembrane alpha helix with strong structural resemblance to phospholamban and sarcolipin—direct interactors with SERCA in the sarcoplasmatic reticulum membrane—co-localises with SERCA and regulates Ca^2+^ handling by inhibiting SERCA pump activity [3].

As already mentioned for *ANRIL*, physiologically important SNPs are not restricted to protein-coding genes. Yang et al. identified a novel mutation on chromosome 2 (rs3819316 C >T) in the lncRNA *Reg1cp* that is associated with elevated bone mass. Mutant *Reg1cp* increased the formation of the CD31^hi^Emcn^hi^ endothelium in the bone marrow to stimulate angiogenesis during osteogenesis. They identified Krüppel-like factor 3 (KLF3) as a protein interaction partner of mutant, but not wild-type *Reg1cp* in human microvascular ECs, and showed that binding of KLF3 to its downstream target JUNB is reduced by mutant *Reg1cp*. Endothelial-specific Klf3 knockout mice also had increased CD31^hi^Emcn^hi^ endothelium and bone formation [73].

## Cancer development and progression

Given the expression of lncRNAs across all tissues and their involvement in fundamental cellular processes, it is unsurprising that lncRNAs have been linked to cancer development and progression. Cancer is a highly complex disease whose hallmarks include, among others, immune evasion, genome instability, angiogenesis and sustained proliferation [23, 24]. Databases such as Lnc2Cancer [19] and Cancer LncRNA Census [65] list lncRNAs already identified to be involved in cancer.

The lncRNAs *PANDA*, *linc-p21* and LINC-PINT are lncRNAs that function to alter transcription factor activity and binding [13, 29, 47]. One of the main members of the coordinated response to genomic instability and mutations arising from DNA damage is the p53 tumour suppressor protein. Hung et al. sought to discover lncRNAs that impact p53-induced cell-cycle arrest [29] and found that the lncRNA termed *PANDA* (P21-associated ncRNA DNA damage activated) is upregulated by p53. Fibroblasts with *PANDA* knockdown displayed increased DNA damage, and a higher sensitivity to DNA damage–induced apoptosis. Interestingly, *PANDA* interacted with the p53-downstream transcription factor nuclear transcription factor Y subunit alpha (NF-YA), which bound more to its target genes after *PANDA* knockdown. These data suggested that *PANDA*, in response to DNA damage, represses NF-YA target gene binding and thereby blocks apoptosis to promote cell survival. Huarte et al. identified a p53-repressor and pro-apoptotic lncRNA termed *lincRNA-p21* [28]. *lincRNA-p21* functions as a mediator of other p53 gene targets. Independent knockdowns of p53 or *lincRNA-p21* revealed an overlap of 930 differentially regulated genes, 80% of which were returned to normal expression levels after a double knockdown of p53 and *lincRNA-p21*. Mechanistically, *lincRNA-p21* recruited and enabled the localisation of hnRNP-K as a p53-dependent repressor that drives the apoptotic response to DNA damage. Cancer cells often develop the ability to depart the primary tumour and invade other tissues and organs to form metastases. The p53-regulated lncRNA LINC-PINT was shown to act as a tumour suppressor, owing to its ability to reduce the invasive and migratory potential of cancer cells [47]. LINC-PINT overexpression was sufficient to downregulate many genes that drive invasion such as Early Growth Response 1 (EGR1), Phospholipase D1 (PLD1), SERPINE1, Fibronectin (FN1) and Integrin alpha 3 (ITGA3), with the upstream regulator identified as β-catenin. The mechanistic function of LINC-PINT is to recruit PRC2 to these invasion-related genes to repress their transcription.

LncRNA TERRA is recruited to telomeres where it stabilises protein-telomere interactions [5]. Telomere shortening determines the replicative potential of cells [72]. Telomeres are repetitive sequences capping the ends of chromosomes; these become shorter with each cell division, eventually losing the ability to protect the chromosome ends and inducing replicative senescence. Cancer cells often have higher levels of telomerase which can lengthen the telomeres and thereby circumvent telomere-induced replicative senescence, important to maintain cancer cell replicative immortality [59]. A highly conserved lncRNA termed TERRA (telomeric repeat-containing RNA) is transcribed from telomeres and acts in *trans* as indicated by its ability to regulate telomeres on other chromosomes in addition to that from which it was transcribed. TERRA is recruited to telomeres and stabilises the interaction of proteins with telomeres. As well as regulating telomerase directly, TERRA can also form RNA-DNA hybrid structures known as R-loops at telomeres to induce homologous recombination and delay senescence. This has been proposed as the mechanism by which telomeres are preserved in the 10% of cancers that do not have elevated telomerase levels [5].

*PCAT19* (prostate cancer associated transcript 19) is another example of a lncRNA whose SNPs have been linked to pathophysiological effects (Fig. 4D). The gene encoding lncRNA *PCAT19* contains a single-nucleotide polymorphism (SNP) that differentially regulates the expression of a short and long isoform of *PCAT19* [27]. The presence of the SNP variant decreases the binding of transcription factors NKX3.1 and YY1 to the *PCAT19*-short promoter that switches to an enhancer and upregulates the *PCAT19*-long isoform. This long isoform of *PCAT19* binds hnRNPAB to activate the transcription of genes involved in cell cycle and the growth and metastasis of prostate cancer tumours. Sustained proliferation is one of the central characteristics of cancer cells.

## Conclusion and outlook

The remarkable progress in the field of lncRNA research has provided evidence that lncRNAs are highly relevant for physiology, development and behaviour. As such, lncRNAs are implicated in a number of diseases, often by fine-tuning gene regulation. Due to their extensive number of features, functions and expression patterns, there is no doubt that lncRNAs offer a great potential and perspective for future interventions such as tissue regeneration and personalised medicine. LncRNA research could lead to innovations that engineer synthetically active lncRNAs for RNA-based therapeutics.

As is the case for protein-coding genes or other ncRNAs, lncRNAs are more than just biomarkers; they are potentially highly specific therapeutic targets [69]. Antisense oligonucleotides targeting natural antisense transcripts (NATs) are an interesting option and showed promising preclinical results. These so-called antagoNATs were used for gene reactivation in the central nervous system and upregulated brain-derived neurotrophic factor and the healthy allele of sodium voltage-gated channel alpha subunit 1 [69]. Not only does this underline the fundamental roles that lncRNAs can play, but it also highlights their potential druggability in many disease scenarios.

Only a relatively small fraction of lncRNAs has been studied so far with the physiological roles of the majority of lncRNAs remaining unknown. Current research is just beginning to examine whether lncRNA genomic loci are functionally independent of the RNA transcript and whether small peptide-coding RNAs add an additional layer of regulation to the system. This raises important questions surrounding the multiple functions of lncRNAs, e.g. on genomic locus-, transcript- and peptide-levels. Many lncRNAs are species- and tissue-specific, indicating that the role of transcriptional activities around the lncRNA loci, the chromatin architecture and regulatory sequence elements within or closely attached to lncRNA gene bodies could be important. Additionally, the structural features of lncRNAs need to be considered. Future studies will also reveal the physiological importance of lncRNA-containing phase-separated condensates, RNA modifications and certain lncRNA isoforms at the single-cell level. The classification of this large group of ncRNAs will also be simplified once a more complete picture of lncRNA biology exists. This could be based on their functionality, processing, structural features and interaction with other RNAs, DNA or proteins. Here, lncRNA databases should take centre stage.

There is no doubt that the progress of lncRNA research will lead to a better understanding of physiological and pathophysiological processes, their fine-tuning and epigenetic control, which can then be used interdisciplinarily to advance therapies and to improve disease outcome.

## Glossary

Alu elements   Short stretches of repetitive DNA that act as transposable elements (mobile genetic elements that can move around the genome). Alu elements are located across the genome and are believed to be functionally relevant, particularly in the generation of new genes in the process of evolution.

*cis*-acting lncRNAs   lncRNAs that regulate gene activity at the same locus from which the lncRNA is transcribed.

Functional elements   Regions of DNA that have a known regulatory function such as promoter regions where transcription factors bind to initiate gene transcription; and enhancers where molecules bind to potentiate the activation of an associated gene.

Histone modifications   Covalent post-translational modifications of the histone proteins that comprise the nucleosome (the basic functional unit of chromatin). Histone modifications include acetylation, methylation, phosphorylation, sumoylation and ubiquitylation to name a few and can regulate the transcriptional state of a gene.

m7G 5′ cap   A methylated guanine nucleotide with a triphosphate linkage on the 5’ end of primary RNA transcripts. This 7-methylguanylate (m7G) functions in nuclear export, intron excision, inhibition of degradation and translation in the case of mRNAs.

Oligonucleotide   Short stretches of DNA or RNA that have a wide range of applications in molecular biology research.

Poly-A tail   Stretch of RNA consisting exclusively of adenine bases. Important for RNA stability, termination of transcription, nuclear export and translation.

R-loop   Three-stranded DNA:RNA structure consisting of two antiparallel DNA strands and an RNA strand. R-loops are involved in gene regulation and genome stability.

SNP   SNPs (single-nucleotide polymorphisms) are substitutions of a single nucleotide in the genome. The majority of SNPs have no functional consequence but some can influence gene regulation and ultimately disease development, depending on their precise location.

Splicing   The process whereby introns are removed from a primary RNA transcript and the exons joined to form the mature RNA transcript.

*trans*-acting lncRNA   lncRNAs that leave their site of transcription to function elsewhere in the cell.

Triplex   Three-stranded oligonucleotide structures of DNA:DNA:RNA in which the RNA occupies the major groove of the DNA and binds through Hoogsteen pairing with the purines of the Watson-Crick basepairs.
